# Enteroviruses from Humans and Great Apes in the Republic of Congo: Recombination within Enterovirus C Serotypes

**DOI:** 10.3390/microorganisms8111779

**Published:** 2020-11-13

**Authors:** Inestin Amona, Hacène Medkour, Jean Akiana, Bernard Davoust, Mamadou Lamine Tall, Clio Grimaldier, Celine Gazin, Christine Zandotti, Anthony Levasseur, Bernard La Scola, Didier Raoult, Florence Fenollar, Henri Banga-Mboko, Oleg Mediannikov

**Affiliations:** 1 IHU Méditerranée Infection, CEDEX 05, 13005 Marseille, France; amoninestin@gmail.com (I.A.); hacenevet1990@yahoo.fr (H.M.); bernard.davoust@gmail.com (B.D.); laminetall30@gmail.com (M.L.T.); clio.grimaldier@ap-hm.fr (C.G.); celine.gazin@ap-hm.fr (C.G.); christine.zandotti@ap-hm.fr (C.Z.); anthony.levasseur@univ-amu.fr (A.L.); bernard.la-scola@univ-amu.fr (B.L.S.); didier.raoult@gmail.com (D.R.); florence.fenollar@univ-amu.fr (F.F.); 2 IRD, SSA, APHM, VITROME, Aix-Marseille University, CEDEX 05, 13385 Marseille, France; 3 Faculté des Sciences et Techniques, Université Marien NGOUABI, Brazzaville, Congo; 4 IRD, AP-HM, MEPHI, Aix Marseille University, 13385 Marseille, France; 5 PADESCA Laboratory, Veterinary Science Institute, University Constantine 1, El Khroub 25100, Algeria; 6 Laboratoire National de Santé Publique, Brazzaville, Congo; jakiana2000@yahoo.fr; 7 Ecole Nationale d’Agronomie et de Foresterie, Université Marien NGOUABI, Brazzaville, Congo; hbangamboko@gmail.com

**Keywords:** enterovirus, non-human primates, humans, genetic recombination, emergence, Republic of Congo

## Abstract

Enteroviruses (EVs) are viruses of the family Picornaviridae that cause mild to severe infections in humans and in several animal species, including non-human primates (NHPs). We conducted a survey and characterization of enteroviruses circulating between humans and great apes in the Congo. Fecal samples (N = 24) of gorillas and chimpanzees living close to or distant from humans in three Congolese parks were collected, as well as from healthy humans (N = 38) living around and within these parks. Enteroviruses were detected in 29.4% of gorilla and 13.15% of human feces, including wild and human-habituated gorillas, local humans and eco-guards. Two identical strains were isolated from two humans coming from two remote regions. Their genomes were similar and all genes showed their close similarity to coxsackieviruses, except for the 3C, 3D and 5′-UTR regions, where they were most similar to poliovirus 1 and 2, suggesting recombination. Recombination events were found between these strains, poliovirus 1 and 2 and EV-C99. It is possible that the same EV-C species circulated in both humans and apes in different regions in the Congo, which must be confirmed in other investigations. In addition, other studies are needed to further investigate the circulation and genetic diversity of enteroviruses in the great ape population, to draw a definitive conclusion on the different species and types of enteroviruses circulating in the Republic of Congo.

## 1. Introduction

Enteroviruses (EVs) are positive-sense single-stranded RNA viruses and members of the Picornaviridae family. These viruses form a genetically diverse genus infecting humans and many animal species, including non-human primates (NHPs), sheep, cattle and pigs [1,2]. The majority of EV infections are asymptomatic, but they can also cause many serious and sometimes fatal diseases in both humans and animals [2,3,4], as in the case of paralytic polio, one of the most important diseases in humans caused by enteroviruses of the poliovirus group.

The enterovirus genus comprises more than 300 serotypes, most of which are known to infect humans, and the most important belong to the group of coxsackieviruses, echoviruses, polioviruses and rhinoviruses. Historically, enteroviruses were originally classified according to their antigenic and pathogenic properties in humans and laboratory animals. These viruses were previously divided into polioviruses (PV) with three serotypes, coxsackievirus A (CV-A) with 23 serotypes, coxsackievirus B (CV-B) with six serotypes, echoviruses (E) with 28 serotypes and enteroviruses 68 to 71 [5,6]. To date, EVs are classified into 12 species, including EV-A to L and rhinoviruses A to C (RV-A, B and C) (https://talk.ictvonline.org/taxonomy/).

Enteroviruses infecting humans belong to species EV-A to EV-D, while infections in animals are mainly caused by EV-E to EV-G species in cattle and pigs and EV-H and EV-J species in NHPs [7,8,9]. The great genetic diversity observed in enteroviruses is linked to several factors, including frequent recombination phenomena and the high mutation rates in their genome. Indeed, it is well-known that genetic recombination contributes to genetic variation and evolution of enteroviruses, thus promoting the emergence and spread of new strains [10,11,12]. For example, the majority of circulating vaccine-derived poliovirus strains have been shown to be recombinant, consisting of either wild poliovirus strains with vaccine strains or poliovirus strains with non-polio enterovirus strains, particularly group C enteroviruses, including coxsackievirus A [13,14,15,16].

Currently, several studies have been conducted worldwide on the occurrence and genetic diversity of human enteroviruses. Similarly, different genotypes have been reported in NHP groups, including African great apes, and their relationships with human enteroviruses have been demonstrated [7,17,18,19,20,21,22]. Studies in Central African countries have shown the presence in wild and captive gorillas and chimpanzees of enteroviruses closely related to those circulating in humans, suggesting possible interspecies transmission between humans and great apes, two genetically similar species [19,20,21].

In addition to the already known human EV species, other typically simian EVs have also been reported within ape species, which could represent a potential source for the emergence of new human diseases [21,22]. Despite the clear evidence of widespread occurrence of EVs in the human and ape populations in Africa and the rest of the world, there are few data available on the occurrence and EV types circulating in the Republic of Congo.

The Republic of Congo is a Central African country with notably high biodiversity and a large forest network organized into protected areas and/or national parks. These areas contain two species of great apes, the central chimpanzees (*Pan troglodytes troglodytes)* and the western lowland gorillas (*Gorilla gorilla gorilla)*. These protected areas, although dedicated to wildlife conservation, also serve as places where humans carry out many activities, which increase human–animal contact and thus promote the exchange of pathogens, especially between humans and the endangered great apes [23]. The objective of this study is to expand data on the circulation and genetic diversity of EVs circulating between humans and great apes in the Republic of Congo (Congo).

## 2. Materials and Methods

### 2.1. Ethics Statement

Research and administrative authorizations for the samples collected from great apes and humans were granted by the Congolese Ministries of Scientific Research and Technological Innovation (n°003/MRSIT/DGRST/DMAST), Forest Economy and Sustainable Development represented by the Congolese Agency for Wildlife and Protected Areas (n°0134/ACFAP-DTS) and the Ministry of Health and Population (N°000220/MSP-CAB-17).

### 2.2. Sample Collection and Study Area

**Figure 1:**
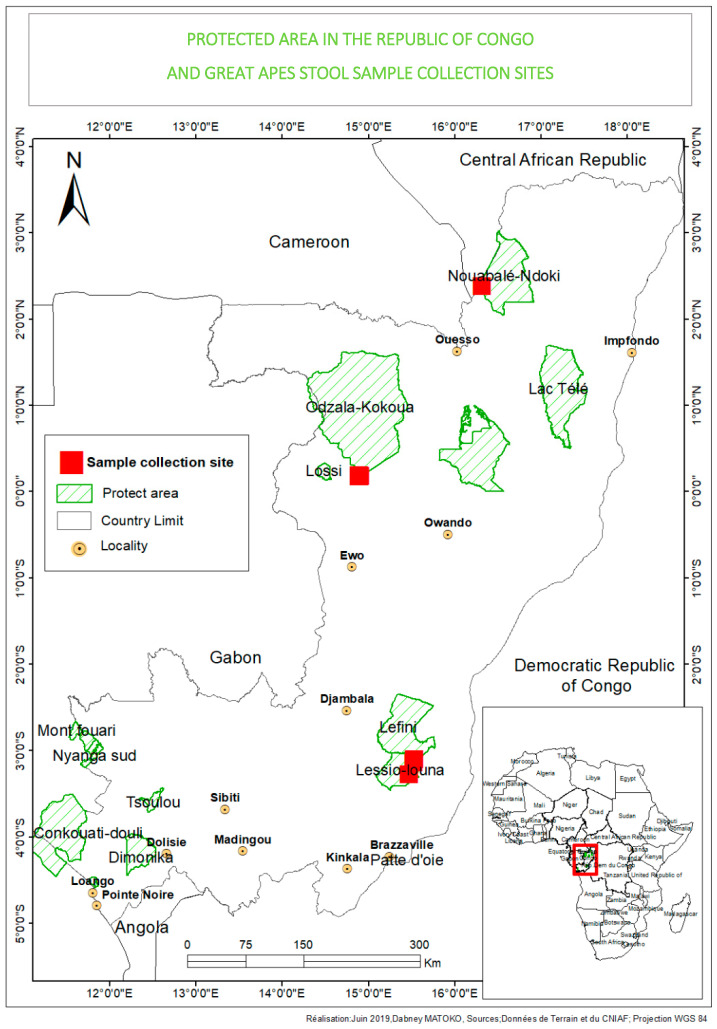
Protected areas in the Republic of Congo and fecal sample collection sites.

**Figure 2:**
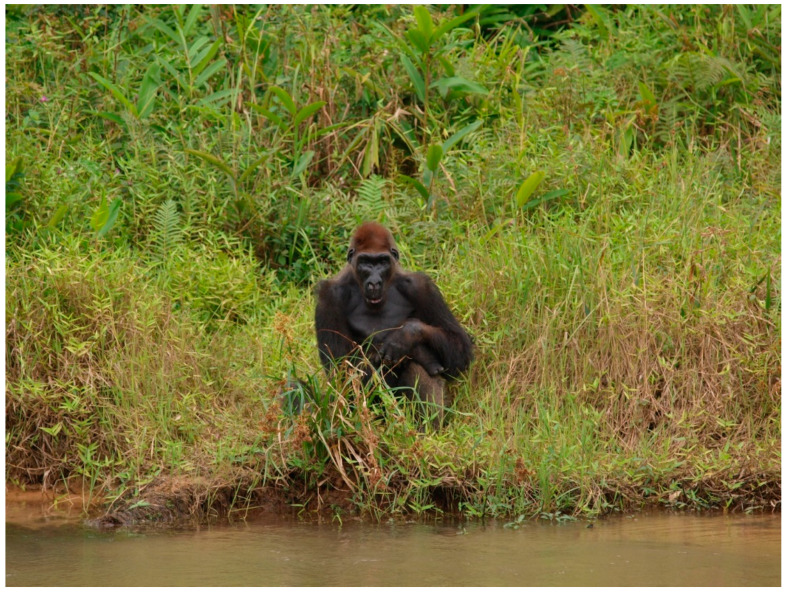
Gorilla (*Gorilla gorilla gorilla*) male individual (Sid) observed on a separate island in front of Abio station of Gorilla Lesio-Louna Nature Reserve. The individual presented with myodystrophy and facial paresis, suggesting a poliomyelitis-like sequelae may be due to the past enteroviral infection.

Fecal samples were collected from NHPs in three national parks in the Congo, as well as from local human populations and healthy eco-guards living in proximity with animals. A total of 24 NHP fresh fecal samples were collected from western lowland gorillas (*Gorilla gorilla gorilla*) living in or not in cohabitation with humans and with wild central chimpanzees (*Pan troglodytes troglodytes*). Eleven of the samples were collected in the Odzala-Kokoua National Park (OKNP) (1.3206°″ N, 14.8455°″ E), 12 samples in the Gorilla Lesio-Louna Nature Reserve (GLLNR) (2°58′33.1″ S, 15°28′33.4″ E) and one sample in the Nouabalé-Ndoki National Park (NNNP) (2.5857°″ N, 16.6291°″ E) (Figure 1). During sample collection in the Lesio-Louna Nature Reserve, we observed an individual named Sid who was kept isolated on a separate island in front of Abio station of GLLNR; we noticed evident marks of myodystrophy and paresis on the face and, probably, legs (Figure 2). For animals living in the wild, stool samples were collected around night nests, feeding sites and places where primates were observed by park guards. For each sample, estimated time of fecal decomposition, and morphological and/or physical appearance were recorded. Provisional identification of ape species was carried out in the field based on such clues as the type of nest near which the feces were found, footprints and texture of the feces.

Human samples were collected after obtaining verbal consent from participants due to their low literacy. A total of 38 samples were collected, including 35 local residents of Mbomo village located within the OKNP, and three were eco-guards of the GLLNR. All great ape samples maintained in absolute alcohol and those kept fresh, as well as human samples, were first stored at −20 °C at the National Public Health Laboratory in Brazzaville before being transported from the Congo to the Institut Hospitalo-Universitaire (IHU) Méditérannée Infection in Marseille for analysis.

### 2.3. RNA Extraction and Molecular Detection of Enteroviruses by RT-qPCR

The extraction of viral RNA was performed using the Qiagen Virus Mini Kit^®^ v2.0 (Qiagen, Courtaboeuf, France) on BIOROBOT EZ1 (Qiagen), according to the manufacturer’s instructions. RNA was extracted from a volume of 190 µL of fecal sample supernatants with 10 µL of internal control, a mixture of the T4 and MS2 phages, and internal controls of viral DNA and RNA [24].

Enterovirus screening was performed by panenterovirus RT-qPCR targeting the 5′-UTR region using a LightCycler Multiplex RNA Virus Master kit (Roche Diagnostics^®^, Mannheim, Germany) as previously described [3]. RT-qPCR reactions were performed using the CFX96 Real-Time system (Bio-Rad Laboratories, Foster City, CA, USA). The thermal cycling conditions included 10 min for reverse transcription at 50 °C, denaturation at 95 °C for 30 s followed by 45 amplification cycles of 5 s at 95 °C and 30 s at 60 °C. Negative (water) and positive controls were included in each reaction.

### 2.4. Molecular Characterization of Enteroviruses and Phylogenetic Analysis

Positive samples by RT-qPCR were subjected to nested RT-PCR targeting the VP1 coding region and the 5’-UTR of enteroviruses using the primers OS1-TTAAAAACAGCCTGTGGTTG and OAS614-ATTGTCACCATAAGCAGCCA for the first amplification (PCR1) and then the primers IS66-GGNAYYYTWGTRCGCCTGTTTTTT and IAS564-CACCCAAAAGTAG TCGGTTCCC for the second amplification (PCR2), as previously described [25].

PCR1 was performed using the SuperScript One-Step RT-PCR with the Platinum Taq kit (Life Technologies) in a final volume of 25 µL, consisting of 5 µL of RNA extract, 12.5 µL of Invitrogen Buffer 2X, 0.5 µL of SuperScript II enzyme (Life Technologies), 6 µL of water and 0.5 µL of each primer (20 μM concentration). The SuperScript II enzyme was activated by incubation at 50 °C for 30 min and 94 °C for 2 min followed by a 40-cycle amplification step at 95 °C for 30 s, 52 °C for 30 s, 72 °C for 30 s and finally a 7 min elongation step at 72 °C. After that step, 1 µL of PCR1 was added to the reaction mixture to perform PCR2. PCR amplification was performed in a reaction mixture of 25 μL containing 12.5 μL of 2X ampliTaq Gold^®^ 360 Master Mix (Applied Biosystems), 0.75 μL of each, 10 μL of sterile water and 1 μL of the PCR1 products. The thermal conditions used in amplification were 15 min at 95 °C for the first denaturation followed by 30 cycles with a denaturation step at 95 °C for 30 s, primer hybridization at 55 °C for 40 s and an elongation step at 72 °C for 30 s followed by a final extension at 72 °C for 5 min. The PCR2 products were visualized on 1.5% agarose gel.

Obtained amplicons of approximately 495 bp were purified using NucleoFast 96 PCR plates (Macherey-Nagel EURL, Hoerdt, France), as per the manufacturer’s instructions, and sequenced. Sequencing was performed using the Big Dye Terminator Cycle Sequencing Kit (Perkin Elmer Applied Biosystems, Foster City, CA, USA) with an ABI automated sequencer (Applied Biosystems). The obtained sequences were analyzed with ChromasPro 1.7.7 software and compared with the sequences of known human and simian EV strains available in the GenBank database (http://www.ncbi.nlm.nih.gov). All sequences were aligned with MEGA7 (https://www.megasoftware.net/), and a phylogenetic tree was constructed using neighbor joining methods. Bootstrap analyses were conducted using 1000 replicates.

### 2.5. Cell Culture and Viral Isolation

Virus isolation was performed with PCR-positive NHP and human samples. Suspensions of treated stool were inoculated onto MA104 and MRC5 cells for 14 days, as previously described [26]. Infected cells were monitored using RT-qPCR, as mentioned above, and were observed under the microscope at day 0 and day 14 for the appearance of cytopathogenic effects. Virus isolates were first characterized (RT-PCR) using the same molecular approaches described above and then subjected to next-generation sequencing (NGS) for whole-genome analysis.

### 2.6. Complete Genome Amplification of EVs

Whole-genome analysis of enteroviruses was performed on culture isolates and RT-PCR-positive stool samples using NGS. To this end, RNA extracts obtained from enterovirus-positive fecal samples and culture isolates were subjected to cDNA synthesis using random hexameric primers and MultiScribe Reverse Transcriptase (Life Technologies) according to the manufacturer’s instructions. The generated single-stranded cDNA was then subjected to Klenow enzyme treatment to switch from single-stranded to double-stranded DNA. The obtained product was quantified by a Qubit assay with a high sensitivity kit (Life Technologies, Carlsbad, CA, USA) to 0.2 ng/µL. Genomic DNA was next sequenced on MiSeq Technology (Illumina Inc., San Diego, CA, USA) with the paired end strategy and was barcoded to be mixed with 5 other genomic projects prepared with the NextEra XT DNA Sample Prep Kit (Illumina). To prepare the paired end library, dilution was performed to require 1 ng of each genome as input to prepare the paired end library. The “tagmentation” step fragmented and tagged the DNA. Then, limited cycle PCR amplification (12 cycles) completed the tag adapters and introduced dual-index barcodes. After purification on AMPure XP beads (Beckman Coulter Inc., Fullerton, CA, USA), the libraries were then normalized on specific beads according to the NextEra XT protocol (Illumina). Normalized libraries were pooled into a single library for sequencing on the MiSeq platform. The pooled single strand library was loaded onto the reagent cartridge and then onto the instrument along with the flow cell. Automated cluster generation and paired end sequencing with dual-index reads were performed in a single 39 h run in 2 × 250 bp. The total information of 62,458 Gb was obtained from a 614 K/mm^2^ cluster density, with a cluster passing quality control filter of 96.9. Within this run, the index representation for enterovirus was determined to index 16.45% for Ibou002 and 30.49% for Mbo044 strains. The 12,473,498 paired end reads were filtered according to the read qualities.

### 2.7. Bioinformatic Analysis NGS

The quality of the sequence data obtained was controlled using CLC Genomics Workbench v7 (https://www.qiagenbioinformatics.com/products/clc-genomics-workbench/) and the assembly was performed by SPADES V3.13.0 [27]. Therefore, we mapped all sequences to the nearest reference to produce consensus sequences to ensure consistency in mapping methodology between high and low coverage samples using the bioinformatics tool CLC. Sequences were trimmed with MiSeq and Trimmomatic [28] software, whereas untrimmed data were processed only using MiSeq software. To decrease assembly gaps, we used GapCloser software [29]. Scaffolds that have a nucleotide number <800 (bp) and scaffolds that have a depth value lower than 25% of the mean depths were removed. The best assembly was selected using different criteria (number of scaffolds, N50, number of N). Finally, the genes were analyzed using a homology search engine, the local BLAST [30,31]. The sequences were aligned using MUSCLE with default parameters. Phylogenetic inferences were obtained using the maximum likelihood method and MEGA software (https://www.megasoftware.net). Bootstrap values obtained by repeating the analysis 1000 times to generate a majority consensus tree are indicated at the nodes, and tree visualization was performed with itol (https://itol.embl.de).

The whole genomes of EV-A to D species available in GenBank with Ibou002 and Mbo044 genomes were analyzed. Phylogenetic trees were constructed with complete sequences from the VP1, VP2, VP3, VP4, 5’-UTR and 3D regions of EVs in MEGA7.

### 2.8. Identification of Great Ape Species

In order to exclude all non-great ape samples and those collected from the same individual, microsatellite analysis of the great ape major histocompatibility complex class I (MHC-I) was performed on all ape fecal samples. PCR targeted the exons 2 and 3, which encode the most polymorphic and functionally relevant parts of the MHC-B gene, as previously described [32,33,34]. The DNA used was extracted from fecal samples according to the protocol described before [35,36], using the EZ1 DNA Tissue Kit (Qiagen).

## 3. Results

### 3.1. Origin of Feces and Differentiation of Great Ape Individuals

We confirmed the origin of feces in 23/24 (95.8%) ape samples. One wild gorilla fecal sample for which the MHC gene sequences were not obtained was excluded from analysis. Of 23 confirmed samples, 22 were identified as being from 17 gorilla individuals and 1 other sample from a chimpanzee individual. Of the gorilla individuals, 12 were from GLLNR gorillas, 1 was from NNNP and 4 individuals were from 9 fecal samples from great apes in OKNP. The chimpanzee individual was identified from feces collected in KNNP (Table S1).

### 3.2. Molecular Detection and Typing of Human and Great Ape Enteroviruses

Enteroviruses were detected in 10 stool samples, including 29.4% (5/18) and 13.15% (5/38) from gorilla and human individuals, respectively (Table S2). No enterovirus was detected in chimpanzee. These enteroviruses were primarily detected in the local human population of Mbomo village (11.4%), eco-guards from GLLNR (33.3%), gorillas cohabiting with humans from GLLNR (16.7%) and gorillas non-habituated to humans from NNNP and OKNP (60%).

**Figure 3:**
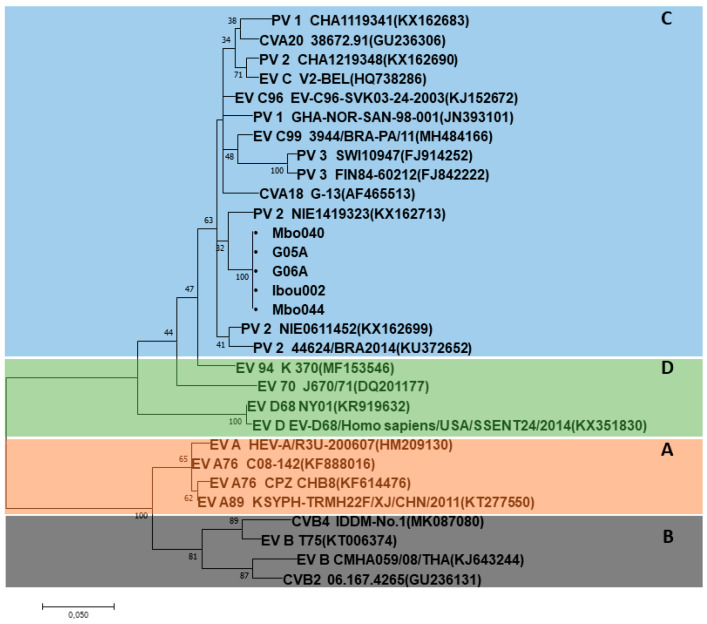
Phylogenetic tree based on the 5’-UTR partial sequences of the human enterovirus serotypes A–D (indicated by colors and letters A–D) and the enterovirus (EV) isolates in the present study (indicated by black circles at the beginning). Isolates form gorillas or from humans, highlighted with black circles, are identical in each other and they clustered with poliovirus (PV)2 (KX162714) of the EV-C.

Characterization of EVs based on the 5’-UTR partial gene was successfully obtained for two of the five gorilla samples and three of the five human-positive samples. The obtained sequences from gorilla (G05A and G06A) and human (Ibou002, Mbo040 and Mbo044) samples were identical to each other and showed 96% identity with PV2_NIE1519322 (KX162714) from Nigeria and 94% with PV1_RC2010-45 (JF838278) from the Congo belonging to EV-C (Figure 3). Only one human sequence was obtained for the VP1 partial gene, which exhibited 86% identity with CV-A13_USA/Ca98-10615 (DQ995644) and CV-A13_BAN00-10562 (DQ995643) isolated from the USA and Bangladesh, respectively, from EV-C.

### 3.3. Isolation and Genomic Analysis of Enterovirus Strains

Virus isolation yielded two strains only in human feces, including one individual from the Mbomo locality (Mbo044) and one eco-guard from the Iboubikro gorilla site in the GLLNR (Ibou002). Genomes were deposed in GenBank under accession numbers LR796218.1 and LR796210.1 for Mbo044 and Ibou002, respectively.

The Mbo044 and Ibou002 genomes were almost identical to each other and shared 99.8% identity. The total length of each genome was 7230 bps. Each strain contained all P1, P2 and P3 regions and all genes were present in the genomes of enteroviruses. Their genomic organization was identical to that of the whole genomes of known enteroviruses without any particular organization difference with the previously described enteroviruses [21]. The P1 region consisted mainly of four structural proteins, VP4 (207 bps), VP2 (813 bps), VP3 (714 bps) and VP1 (924 bps). However, the P2 and P3 regions, which include all the non-structural proteins, consisted of genes 2A (444 bps), 2B (291 bps), 2C (922 bps), 3A (329 bps), 3B (66 bps), 3C (549 bps) and 3D (1263 bps). In addition to these three regions (P1, P2 and P3), a 5′-UTR region (708 bps) was also present in both genomes. The two strains were compared gene by gene with other reference strains available in the GenBank (Table 1).

**Figure 4:**
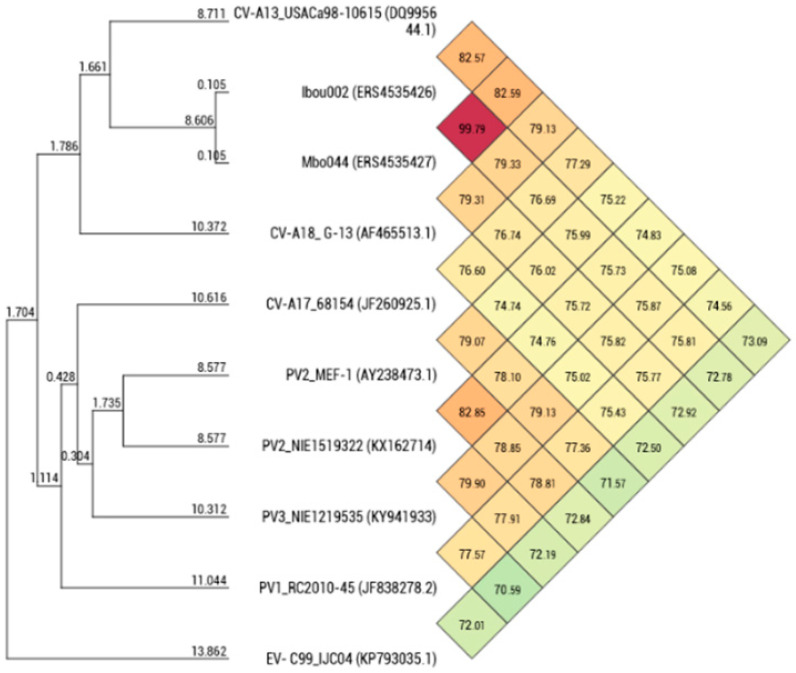
Heat map generated with OrthoANI values calculated by OAT software between Mbo044 and Ibou002 strains and other closely related EV-C species with standing in nomenclature.

The degree of genomic similarity of Mbo044 and Ibou002 (closely related enteroviruses C) was estimated by OrthoANI software [37]. Values among closely related species ranged from 70.59% between *PV3_NIE1219535 (KY941933)* and EV-C99_IJC04 (KP793035) to 82.85% between *PV2_MEF-1 (AY238473)* and PV2_NIE1519322 (KX162714). When the isolates were compared to these closely related species, values ranged from 72.78–72.92% (Ibou002–Mbo044) with EV-C99_IJC04 (KP793035) to 82.57–82.59% (Ibou002–Mbo044) with *CV-A13_USACa98-10615 (DQ995644)* (Figure 4).

**Figure 5:**
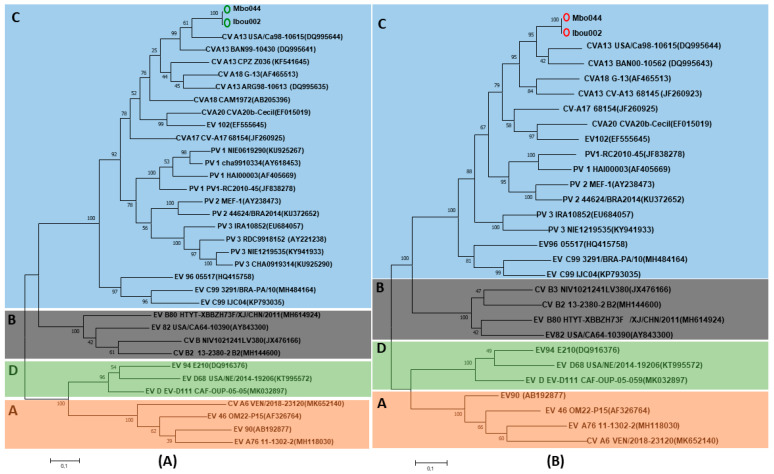
Phylogenetic tree based on the complete VP1 (**A**) and VP2 (**B**) sequences of the human enterovirus serotypes and EV strains in this study. Isolates in the present study are indicated in green (VP1) and red (VP2) circles while EV-C isolates previously described in the Congo and elsewhere are in blue.

Phylogenetic analysis of Mbo044 and Ibou002 whole genomes, as well as complete genes of the structural capsid protein regions VP1–VP4, showed that both strains were identical to each other and grouped with coxsackievirus A serotypes, a member of enterovirus C (EV-C). In Blastn, the Ibou02 and Mbo044 strains were 82.8% close to CV-A13_USA/Ca98-10615 (DQ995644) and CV-A13_BAN00-10562 (DQ995643) isolated from humans in the USA and Bangladesh, respectively. However, in the 5’-UTR and 3D regions, the two strains were 93.5% and 85.8% similar to poliovirus 2 NIE1519322 (KX162714) from the stool of a human in Nigeria and 91.7% and 86.4% to poliovirus 1 PV1_RC2010-45 (JF838278) from humans in the Congo, which is also a member of the EV-C, respectively (Figure 4 and Figure 5, Figures S1 and S2).

**Figure 6:**
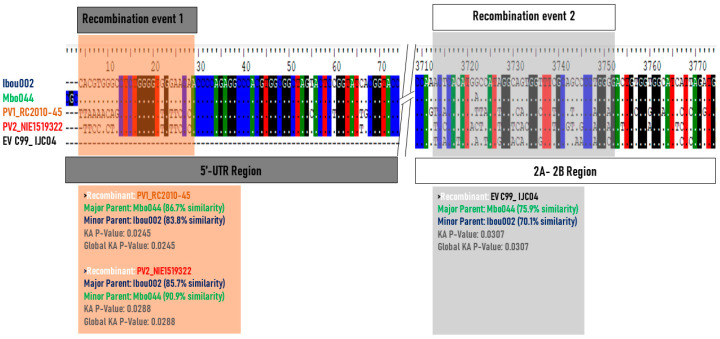
Schematic representation of recombinant regions between Ibou002 and Mbo044 genomes with the poliovirus strains PV1 (JF838278) from Congo and PV2 (KX162714) from Nigeria (PV1 and PV2) and non-poliovirus strain EV-C99 (KP793035), also isolated in Congo, using Recombination Detection Program 4 (RDP4). Recombination localized in the 5′-UTR region (1–690 bps), *p*-value = 0.045 and 0.0288 for PV1 and PV2, respectively, and in 2A and 2B regions (3740–3980 bps), *p*-value = 0.0307 for EV-C99.

**Figure 7:**
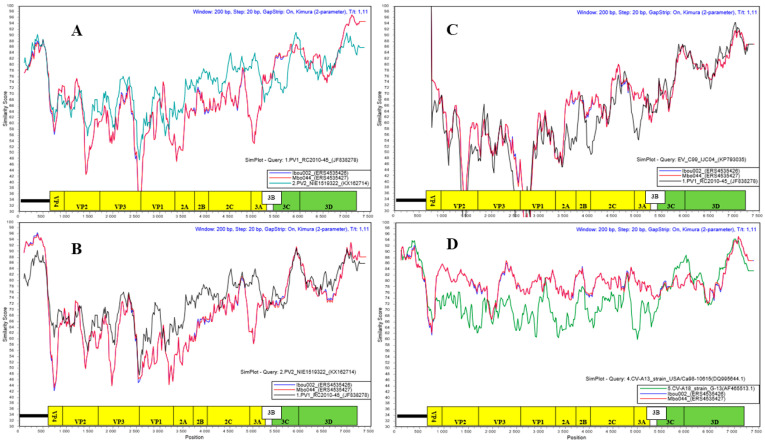
Pairwise comparison analysis of PV1_RC2010-45 (JF838278) (**A**), PV2_NIE1519322 (KX162714) (**B**), EV-C99_IJC04 (KP793035) (**C**) and coxsackievirus (CV)-A13_strain_USA/Ca98-10615 (DQ995644) (**D**) full-length genomes with Ibou002 and Mbo044 genomes of this study. Recombination was analyzed with the SimPlot software, version 3.5.1. Levels of pairwise identities between sequences were determined by using the Kimura (two-parameter) distance method with a 200 nt wide window and a step size of 20 nt.

To determine whether strains Ibou002 and Mbo44 were actually recombinants, their whole genomes were compared to those of five EV-C strains, including one coxsackievirus CV-A13 (DQ995644), three polioviruses (PV1 (JF838278), PV2 (KX162714) and PV3 (KY941933)) and EV-C99 (KP793035) by GENECONV algorithm analysis [38] of the Recombination Detection Program 4 (RDP4) [39]. Recombination events were found between Ibou002 and Mbo044 with the PV1 strain from Congo and PV2 from Nigeria in the 5′-UTR region (1–690 bps) (*p*-value = 0.045 and 0.0288), then in the 2A and 2B regions (3740–3980 bps) with the strain EV-C99, also isolated in Congo (*p*-value = 0.0307). These three EV strains (PV1, PV2 and EV-C99) were thus considered to be potential recombinants with the Ibou002 and Mbo044 strains (Figure 6, Figures S3 and S4). In the recombination with the PV2 strain, the major parent was the Ibou002 strain, although the percentage of similarity was higher with Mbo044 (90.9%) than with Ibou002 (85.7%). On the other hand, Mbo044 was the major parent in the recombination with PV1 and EV-C99 with 86.7% and 75.9% similarity, respectively. No recombination events were observed with the strains CV-A13 and PV3. In addition, recombination was analyzed and confirmed with SimPlot software version 3.5.1 (https://sray.med.som.jhmi.edu/SCRoftware/simplot/). Levels of pairwise identities between the sequences cited above and sequences in the present study were determined by using the Kimura (two-parameter) distance method with a 200 nt wide window and a step size of 20 nt (Figure 7).

## 4. Discussion

The objective of this study was to assess the circulation and genetic diversity of enteroviruses in wild and captive great apes in three national parks in the Congo, as well as that of humans living around and within the parks. Enteroviruses were detected in 29.4% of gorilla individuals and 13.15% of humans. Enteroviruses were characterized and confirmed by RT-PCR/sequencing of the 5’-UTR and/or VP1 region. Two strains were isolated from human samples.

Although discordant in terms of prevalence, these results are concordant with previous studies reporting the circulation of a wide range of enteroviruses in both humans and great ape populations from different African countries and elsewhere [16,19,20,22,40,41,42,43]. Interestingly, the strains and amplified genome fragments from all territories and from humans and NHPs are almost identical. The strains appear to be of clonal origin and circulate on the large territory from Lesio-Luna on the South to Nouabalé-Ndoki, Northern Congo.

Based on the 5’-UTR partial sequences or the VP1 coding region, all enterovirus sequences obtained directly from gorilla and human fecal samples were similar to each other, and they are closely related to EV-C, specifically PV2_NIE1519322 (KX162714). As in many countries around the world, non-polio EV-C and polioviruses have already been reported in humans from the Congo [21,44,45]. Additionally, the presence of HEV-C was reported in a captive chimpanzee with acute flaccid paralysis symptoms living in a sanctuary in the Southern Congo (EV-C99) [21].

Although the gorilla male individual named Sid (Figure 2) was not found to have EV, either by molecular testing or by isolation, we may suggest that highly typical poliomyelitis-like sequelae may be due to past enteroviral infection [44,46]. Further serological studies should be conducted to confirm this hypothesis. However, one of two isolated strains (Ibou002) was recovered from a guard living immediately in front of Sid’s island.

To better characterize the detected enteroviruses, EV strains were isolated from human stool samples. Unfortunately, no viruses were isolated from gorilla fecal samples. This finding could be explained by the degradation of gorilla fecal samples taken from the ground and then preserved in alcohol.

In this study, cell culture for the isolation of enteroviruses was performed with MA104 and MRC5 cells from all human and ape stool samples positive in real-time PCR. Indeed, although these cells are also sensitive to enteroviruses [47], the use of other cell lines that are more sensitive and known to be effective in isolating a certain group of enteroviruses should be considered in order to increase the spectrum of virus isolation in stool. This suggests that the absence of these cell lines in the isolation technique used was probably responsible for the low rate of virus strains isolated in addition to the quality of feces in great apes. For example, high rates of isolation of enteroviruses, particularly enteroviruses of species C (HEV-C), have been reported in most countries with the use of HEp-2c, RD and L20B cell lines [10,11,19,46,48].

Whole genomes of the isolated strains were perfectly identical to each other, and they were similar to CV-A from the EV-C group, particularly CV-A13_USA/Ca98-10615 (DQ995644) and CV-A13_BAN00-10562 (DQ995643), as observed for the VP1, VP2, VP3 and VP4 regions of both genomes. In contrast, in the 5’-UTR and 3D regions, strains Mbo044 and Ibou002 were similar to PV2_NIE1519322 (KX162714) and poliovirus 1 PV1_RC2010-45 (JF838278). Similar to polioviruses, CV-A are members of EV-C, and their circulation and abundance have been widely documented [19,20,40,41,46,49,50]. Coxsackievirus A is a well-known human pathogen and is often responsible for serious and sometimes fatal infections in humans.

The presence of sequences close to those of PV and CV-A in the isolated strains shows that both Ibou002 and Mbo044 strains may derive from a combination of polio and non-polio HEV-C strains, suggesting the possibility of recombination within these two strains. The strains could thus be responsible for the emergence of a new strain of PV or represent a new virus within the HEV-C group. Indeed, most circulating vaccine-derived polioviruses (cVDPVs) have been shown to have mosaic genomes that are recombinant between PV and other HEV-C, mainly CV-A viruses [14,46]. In addition, recombination events were detected between Ibou002 and Mbo044 with the PV1 (JF838278) from Congo and PV2 (KX162714) from Nigeria in the 5’-UTR region (1–690 bps) (*p*-value = 0.045 and 0.0288), followed by the 2A and 2B regions (3740–3980 bps) with the strain EV-C99 (KP793035), which was also isolated in the Congo (*p*-value = 0.0307). These three enterovirus strains were thus considered to be potential recombinants with the Ibou002 and Mbo044 strains (Figure 6, Figures S3 and S4). In contrast, no recombination events were observed with the genome sequences of strains CV-A13 (DQ995644) and PV3 (KY941933). Based on data from the cVDPV literature in outbreak regions, such as Cambodia, Madagascar, Democratic Congo and Nigeria, CV-A, including CV-A13, CV-A17, CV-A20 and CV-A11, have been reported as partners most frequently involved in recombination with PV strains [14,16,41,46,49,51]. In addition to infecting humans and being enteroviruses of human origin, CV-A has also been found in NHPs. A study conducted in Cameroon demonstrated the presence of CV-A13 and CV-A24 in stool samples from captive chimpanzees and gorillas, suggesting the circulation of HEVs in the African great ape populations [19].

Although limited by the number of samples, our study demonstrated, potentially, the presence of the same EV species (EV-C) in both humans and gorillas living in the same biotopes in several regions of the Congo, indicating a possible cross-species transmission between humans and apes and a wide distribution of this strain. The isolated strains possessing identical sequences to two different EV-C types (CV-A13 and PV) suggest the possibility of recombination within EV strains circulating in the Congo. Recombination analysis revealed recombinant events in the Ibou002 and Mbo044 strains with PV1, PV2 and EV-C99. These viruses could also be new viruses within the HEV-C group, in addition to known human viruses. Given the great diversity of these viruses and the continuous observation of new strains generated by their high mutation rates and frequent recombination, systematic surveillance of EVs plays a crucial role in epidemic prevention policies.

Furthermore, it is generally well-known that enteroviruses, like most enteric viruses, have evolved under unfavorable environmental conditions, including thermal stability, acid stability, resistance to radiation as well as to oxidants and proteolytic enzymes, which allow their survival in the environment and facilitate their transmission through multiple environmental pathways (e.g., water, food, aerosols …, etc.). Human enteroviruses have been recovered worldwide from several types of environment, including surface waters, coastal waters, rivers, streams and lakes, groundwater and wastewater [52]. In addition, studies of the effects of temperature on the survival of some enteric viruses, including some types of enteroviruses, indicate that temperature is one of the critical factors affecting their stability, in that the higher the temperature, the faster the loss of viral infectivity [52,53]. Thus, we did not exclude false negative results in the detection of EVs in the present study, which could be due to poor storage conditions of the samples, particularly, insufficient cold chain duration. Indeed, the great ape and human feces samples used were collected in national parks, far from urban centers with difficult access to roads and electricity. This state must be considered in the future studies.

The main limit of the study is the absence of other sequences of the enterovirus detected in great apes. The only sequences obtained in gorillas were just from the amplification of 5′-UTR. However, it is well-known that most of the variability in enteroviruses comes from the P1 region, and it is much more reliable. In addition, the absence of other cell lines in the isolation technique used probably led to a reduction in the rate of strains isolated in the present study. In view of this result, which seems to be insufficient for precise typing of enteroviruses, it is very difficult to conclude on the type of enteroviruses observed in the great apes. Thus, future studies are planned in order to deepen the data on the typing of enteroviruses.

As observed in this study and elsewhere, recombination between poliovirus strains (wild type and vaccine strains) and other enteroviruses must be monitored to prevent and anticipate the emergence of new pathogenic enterovirus strains and any new epidemics. Also, the data from this study provide a basis for the need to monitor and swallow the circulation and genetic diversity of enteroviruses in wild and captive great apes in the Republic of Congo.

## Figures and Tables

**Table 1:** Identity between the genomes of Ibou044 and Mbo02 strains with non-polio enterovirus C and polioviruses. Highest identity is in bold.

Region		Amino Acid Identity (%)							
	Length	CV-A13 (DQ995644)	CV-A17 (JF260925)	CV-A18 (AF465513)	CV-A20 (EF015019)	EV-C99 (KP793035)	PV1 (JF838278)	PV2 (KX162714)	PV3 (KY941933)
5’-UTR	708	**89.24**	**91.27**	**91.13**	**91.40**	-	**85.53**	**93.41**	**91.77**
VP4	207	84.54	84.18	**91.13**	81.59	80.10	79.80	78.17	81.07
VP2	813	82.90	73.13	**83.00**	74.02	72.94	-	74.04	73.43
VP3	714	82.49	76.36	78.11	74.71	74.86	74.43	75.69	74.31
VP1	924	83.03	-	75.87	-	74.22	72.40	69.42	70.51
2A	444	82.17	-	-	72.81	72.21	74.04	73.38	72.54
2B	291	80.07	-	78.56	73.29	72.16	74.38	75.53	75.09
2C	922	**89.23**	-	73.08	-	77.48	76.92	76.87	76.25
3A	329	82.76	81.05	77.97	80.84	80.80	81.93	82.73	82.00
3B	66	**85.79**	**89.83**	-	**88.14**	**89**	**88.14**	**88.52**	**90.16**
3C	549	**85.32**	-	-	**85.98**	**85.06**	**86.16**	**86.25**	**85.61**
3D	1263	**85.10**	-	-	**85.74**	**86.39**	**86.66**	83.29	**85.74**
Mbo044 (LR796218)	7230	**82.80**	77.43	78.91	77.63	77.83	78.57	77.63	77.69
Ibou002 (LR796210)	7230	**82.77**	77.44	78.88	77.58	77.80	78.52	77.64	77.77

## Supplementary Materials

The following are available online at https://www.mdpi.com/2076-2607/8/11/1779/s1. Figure S1: Phylogenetic tree based on the complete VP3 (**A**) and VP4 (**B**) sequences of the human enterovirus serotypes and EV strains. Strains in the present study are indicated in green (VP3) and red (VP4) circles while EV-C isolates previously described in the Congo and elsewhere are in blue. Figure S2: Phylogenetic tree based on the complete 3D (**A**) and 5′-UTR (**B**) region sequences of the human enterovirus serotypes and the EV strains in this study. Isolates in the present study are indicated in green (3D) and red (5’-UTR) circles while EV-C isolates previously described in the Congo and elsewhere are in blue. Figure S3: Schematic sequence display of recombinant regions between Ibou002 and Mbo044 genomes with the poliovirus strains PV1 (JF838278) from the Congo and PV2 (KX162714) from Nigeria (PV1 and PV2) using the Recombination Detection Program (RDP). Recombination located in the 5′-UTR region (1–690 bps) (*p*-value = 0.045 and 0.0288 for PV1 and PV2, respectively). Figure S4: Schematic sequence display of recombinant regions between Ibou002 and Mbo044 genomes with the non-poliovirus strain EV-C99 (KP793035) also isolated in the Congo using the Recombination Detection Program (RDP). The event located in 2A and 2B regions (3740–3980 bps) (*p*-value = 0.0307). Table S1: Distribution of great ape individuals by collection sites. Table S2: Number and prevalence of humans and gorillas infected with enteroviruses.
