# Supplementary material for: Enteroviruses from Humans and Great Apes in the Republic of Congo: Recombination within Enterovirus C Serotypes
Source: Microorganisms. 2020 Nov 13;8(11):1779. doi: 10.3390/microorganisms8111779 (PMC7709013; doi:10.3390/microorganisms8111779)
Supplement: Supplementary file 1 [file microorganisms-08-01779-s001.zip › Table S1.docx]

**Table S1**. Distribution of great ape individuals by collection sites

| Sites | Number of fecal samples | | | Number of individuals | |  |
| --- | --- | --- | --- | --- | --- | --- |
|  | Gorilla | Chimpanzee | Total | Gorilla | Chimpanzee | Total |
| OKNP | 9 | 1 | 10 | 4 | 1 | 5 |
| NNNP | 1 | 0 | 1 | 1 | 0 | 1 |
| GLLNR | 12 | 0 | 12 | 12 | 0 | 12 |
| Total | 22 | 1 | 23 | 17 | 1 | 18 |
