# Supplementary material for: Enteroviruses from Humans and Great Apes in the Republic of Congo: Recombination within Enterovirus C Serotypes
Source: Microorganisms. 2020 Nov 13;8(11):1779. doi: 10.3390/microorganisms8111779 (PMC7709013; doi:10.3390/microorganisms8111779)
Supplement: Supplementary file 1 [file microorganisms-08-01779-s001.zip › Table S2.docx]

**Table S2.** Number and prevalence of humans and gorillas infected with enteroviruses

| Species | Site/Town | Number of individuals |  | Number of infected individuals | Percentage of  infected individuals |
| --- | --- | --- | --- | --- | --- |
| Gorillas co-habiting humans | GLLNR | 12 |  | 2 | 16.67 |
| Gorillas non co-habiting humans | OKNP and NNNP | 5 |  | 3 | 60 |
| Total |  | 17 |  | 5 | 29.41 |
| Human | Lesio-Louna eco-guards | 3 |  | 1 | 33.33 |
|  | Mbomo Local population | 35 |  | 4 | 11.42 |
| Total |  | 38 |  | 5 | 13.15 |
