# Supplementary figures and images for: Enteroviruses from Humans and Great Apes in the Republic of Congo: Recombination within Enterovirus C Serotypes
Source: Microorganisms. 2020 Nov 13;8(11):1779. doi: 10.3390/microorganisms8111779 (PMC7709013; doi:10.3390/microorganisms8111779)

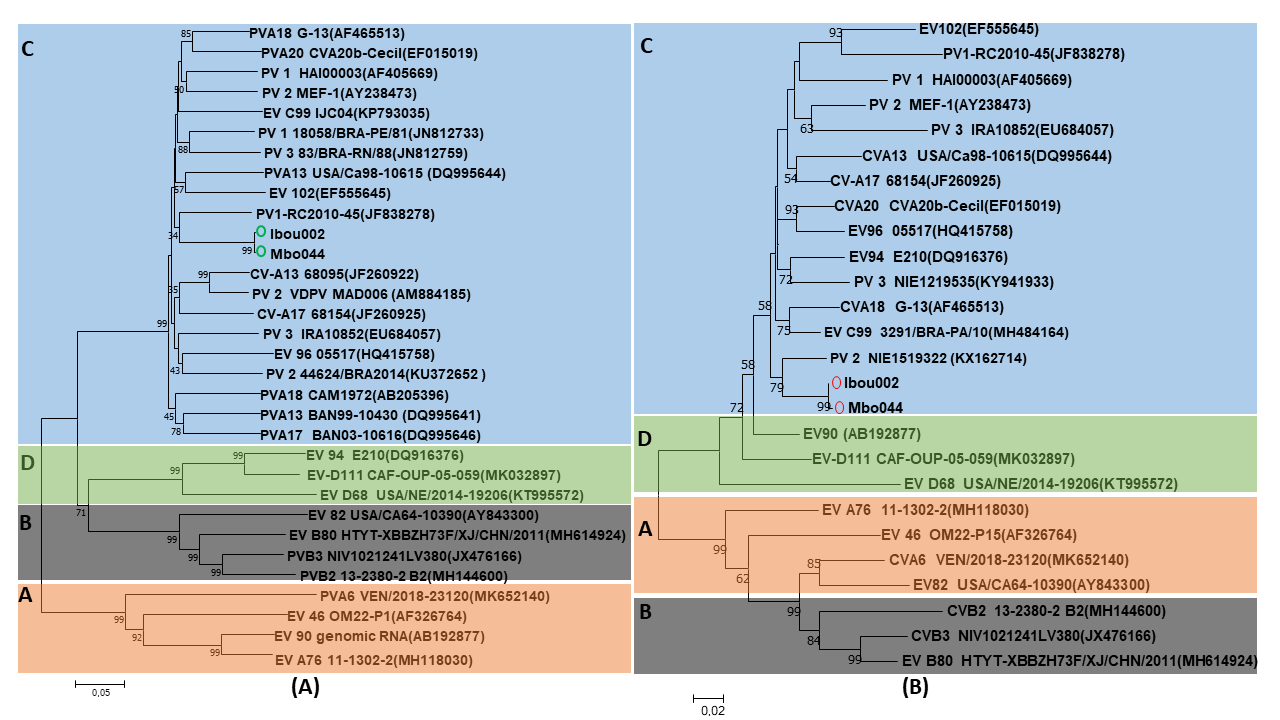

Supplement: Supplementary file 1 [file microorganisms-08-01779-s001.zip › Figure S2.tif]

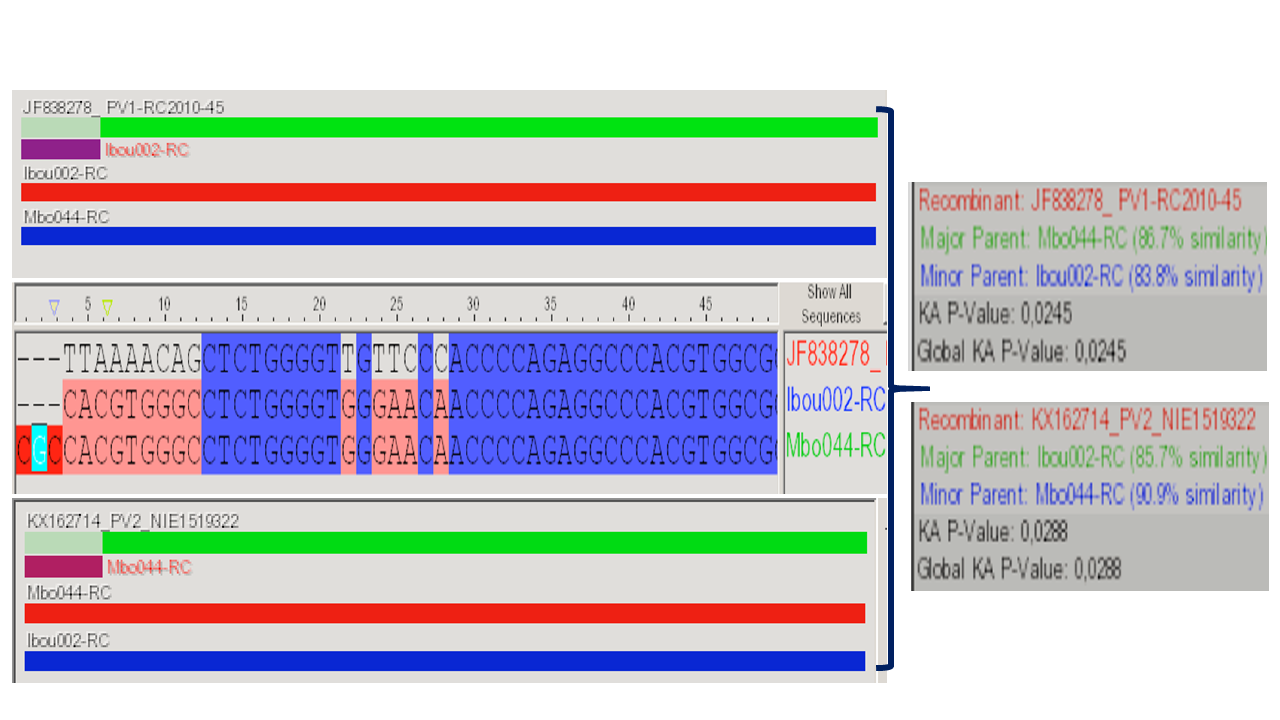

Supplement: Supplementary file 1 [file microorganisms-08-01779-s001.zip › Figure S3.tif]

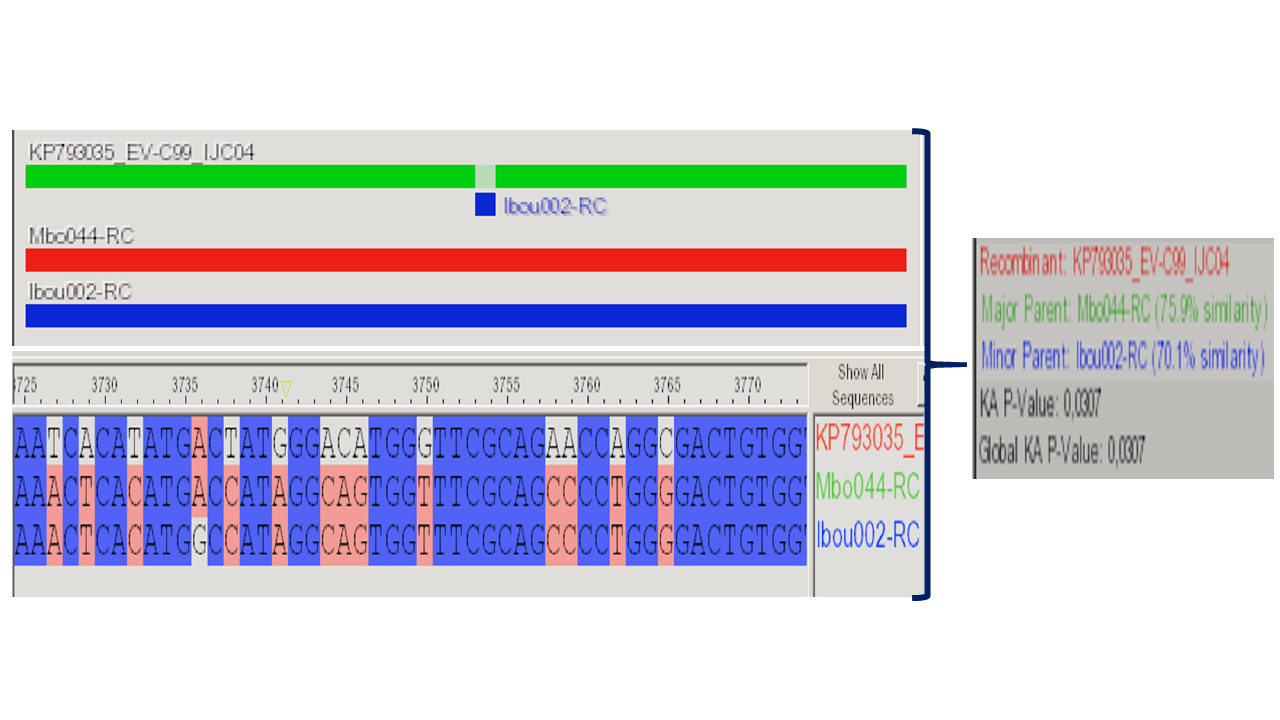

Supplement: Supplementary file 1 [file microorganisms-08-01779-s001.zip › Figure S4.tif]

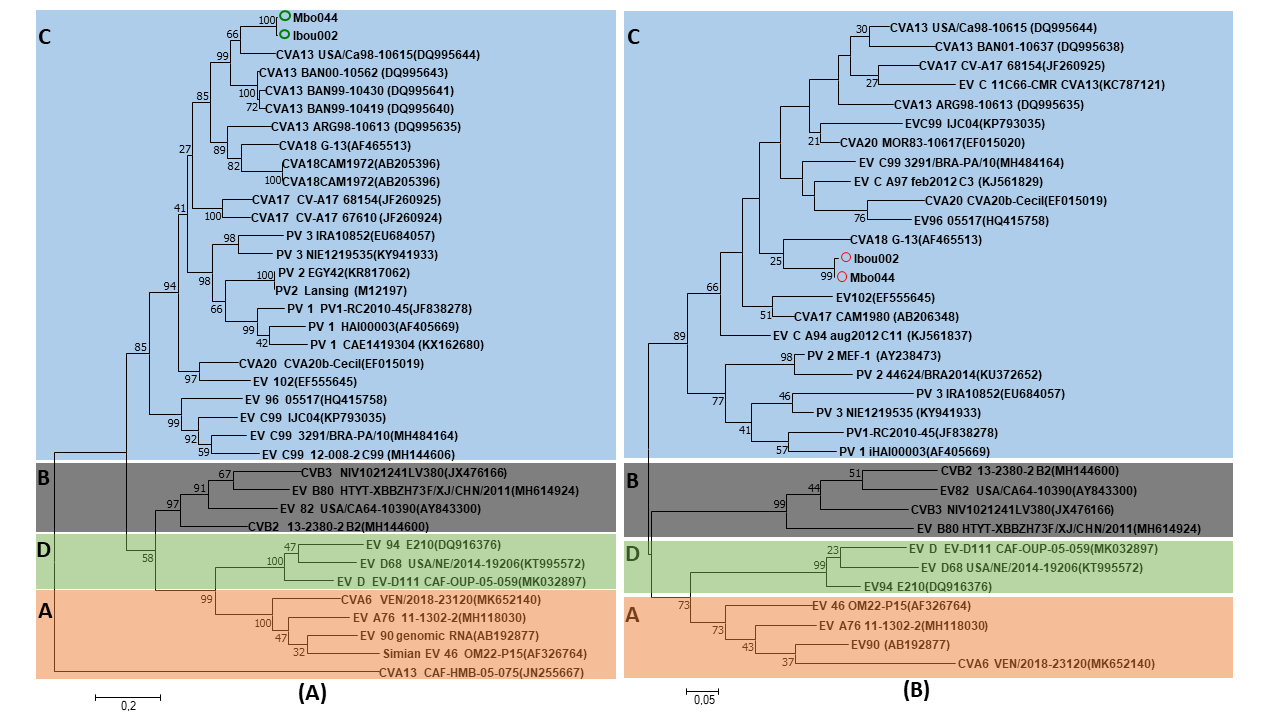

Supplement: Supplementary file 1 [file microorganisms-08-01779-s001.zip › Figure S1.tif]
